# Yoga to improve maternal mental health and immune function during the COVID-19 crisis (Yoga-M _2_ trial): study protocol for a pilot randomized controlled trial.

**DOI:** 10.12688/wellcomeopenres.17729.1

**Published:** 2022-03-23

**Authors:** Rahul Shidhaye, Vidyadhar Bangal, Hemant Bhargav, Swanand Tilekar, Chitra Thanage, Rakhee Suradkar, Kalpesh Game, Vandana Pulate, Sonali Tambe, Vaibhav Murhar, Rahul Kunkulol

**Affiliations:** 1 Senior Research Scientist and Associate Professor of Psychiatry, Pravara Institute of Medical Sciences, Loni, Maharashtra, 413736, India; 2 Visiting Researcher, Department of Health, Ethics and Society, Care and Public Health Research Institute, Maastricht University, Maastricht, The Netherlands; 3 Professor and Head, Department of Obstetrics and Gynecology, Pravara Institute of Medical Sciences, Loni, Maharashtra, 413736, India; 4 Assistant Professor, Department of Integrative Medicine, National Institute of Mental Health & Neurosciences, Bengaluru, Karnataka, 560029, India; 5 Assistant Professor and Research Officer, School of Public Health and Social Medicine, Pravara Institute of Medical Sciences, Loni, Maharashtra, 413736, India; 6 Project Coordinator, Directorate of Research, Pravara Institute of Medical Sciences, Loni, Maharashtra, 413736, India; 7 Yoga Instructor, Directorate of Research, Pravara Institute of Medical Sciences, Loni, Maharashtra, 413736, India; 8 Clinical Research Coordinator, Directorate of Research, Pravara Institute of Medical Sciences, Loni, Maharashtra, 413736, India; 9 Tutor, Department of Pharmacology, Pravara Institute of Medical Sciences, Loni, Maharashtra, 413736, India; 10 Independent Researcher, Independent Researcher, Bhopal, Madhya Pradesh, 462016, India; 11 Professor and Head, Department of Pharmacology, Pravara Institute of Medical Sciences, Loni, Maharashtra, 413736, India

**Keywords:** Yoga, Pregnancy, Stress, Immunity, India

## Abstract

**Background: **Mental health of women is adversely affected during pregnancy. A huge proportion of pregnant women suffer from stress and depression which negatively impacts birthweight and neuro-cognitive development of the fetus. The current crisis due to the COVID-19 pandemic further adds to the stressful situation. Yoga practiced during pregnancy has beneficial effects on improving stress and depression and preliminary evidence suggests that yoga-based interventions can improve immunity. This study aims to examine the feasibility, acceptability, and preliminary efficacy of a **Yoga**-based intervention for maternal **M** ental health and i **M** munity (Yoga-M _2_) in a rural community in India.

**Methods: **The study design will be a single-blind individual randomized parallel group-controlled pilot trial with 1:1 allocation ratio. Adult pregnant women, with gestational age between 12–24 weeks will be randomly allocated to either the Yoga-M _2 _group or the Enhanced Usual Care (EUC) group. Participants in the Yoga-M _2_ arm will attend weekly group yoga sessions for 12 weeks and will be encouraged to practice yoga at home. In the EUC arm, participants will receive a single session of health education. Eligibility of the participants, recruitment, retention-in-care, and study completion rates will be estimated and feasibility of delivering Yoga-M _2 _and acceptability of this intervention by the participants will be assessed. Change in the scores of the Perceived Stress Scale (PSS), EuroQoL 5 Dimensions Score (EQ-5D-5L), Wisconsin Upper Respiratory Symptom Severity Scale (WURSS-21), and serum C-Reactive Protein at three-months post-randomization will be used to assess preliminary efficacy.

**Discussion:** The key outputs of this trial will be a structured intervention manual and evidence about the feasibility, acceptability, and preliminary efficacy of the intervention, establishing the foundation to undertake an explanatory randomized controlled trial to assess efficacy and cost-effectiveness of Yoga-M _2_ intervention.

**Trial registration:** CTRI/2022/01/039701. Prospectively registered with the Clinical Trials Registry of India on 25 January 2022.

## Introduction

### Background

The global crisis due to the novel coronavirus disease (COVID-19) will enter its third year and has claimed more than 5.7 million lives as on 11 February 2022 according to the World Health Organization database. Despite the availability of several vaccines, the new variants (e.g., Omicron) are emerging at regular intervals leading to multiple waves of COVID-19 infections and disruption of economic and social activities. The pandemic may go on for some time into the future, posing a serious challenge to already stretched health care systems in India and other parts of the globe. The disruption due to COVID-19 has severely affected the access and quality of health services delivered to non-COVID patients, especially the vulnerable sections of the society such as pregnant women ^1^. 

During pregnancy, changes in cardiorespiratory and immune system functioning increase the susceptibility of women to severe infection and hypoxic compromise ^2^. COVID-19 during pregnancy increases the risk of preeclampsia, venous thromboembolism, premature fetal membrane rupture, or preterm delivery ^3, 4^. In addition to increased susceptibility to infections, pregnant women are also more vulnerable to psychological stress. Mental health conditions affect pregnant women, especially depression which is quite common during perinatal period. One in four women in South Asia is likely to experience depression during pregnancy, while in India, the pooled prevalence of antenatal depression is 17.7% (95% CI: 11.2%–26.9%) ^5^. Antenatal depression is associated with higher risk of pregnancy-related mortality and morbidity, pre-term delivery, low birth-weight, post-partum depression and adverse neuro-cognitive developmental outcomes in infants, children and adolescents ^6^.

Yoga is a mind-body practice, and its roots can be traced in ancient Indian philosophy. Yoga has been practiced in India for more than 7000 years ^7^. Yoga during pregnancy can be very helpful in prevention and treatment of stress and depression ^8^. A meta-analysis found a positive effect of yoga on antenatal depression ^9^. Another systematic review assessing the effect of yoga on immune system functioning found that yoga can down regulate pro-inflammatory markers and enhance cell-mediated and mucosal immunity. This makes yoga a suitable intervention for individuals suffering from diseases with inflammatory component and those who are at higher risk of getting infections ^10^. There is only one study which has assessed the effect of yoga on immune function during pregnancy ^11^. In this study, yoga-based intervention, which included postures, deep breathing, guided imagery, and deep relaxation was delivered to 48 women during pregnancy, 70 minutes per session, 2 sessions per week for 20 weeks. Pregnant women who practiced yoga had higher immunoglobulin A levels and lower salivary cortisol levels immediately after a yoga session, compared to those who did not practice yoga. In the long-term, salivary immunoglobulin A levels were significantly higher in the yoga group compared to the control group ^11^.

### Rationale for study

The possibility of another COVID-19 wave in India (and other parts of the globe) can’t be entirely ruled out. This necessitates generating evidence for population-based interventions which can improve immune system functioning and reduce the susceptibility of infections in vulnerable groups like pregnant women. Yoga can be practiced by pregnant women as self-care with minimal training. However, there are no studies from India which have assessed the effects of yoga-based intervention on immune system functioning in pregnant women.

### Objectives

The overall aim of this pilot randomized controlled trial is to examine the feasibility, acceptability, and preliminary efficacy of a **Yoga**-based intervention to improve maternal **M**ental health and i **M**munity (Yoga-M _2_).

The primary objectives of this randomized pilot trial are:

1. To estimate participant eligibility, recruitment and retention-in-care and study completion rates.

2. To assess the feasibility of delivering Yoga-M _2 _and acceptability of this intervention by the participants.

3. To assess the preliminary efficacy of Yoga-M _2 _to improve maternal mental health and immune function.

**Trial design**

Single-blind individual randomized parallel group-controlled pilot trial with 1:1 allocation ratio.

## Methods

This protocol follows the Standard Protocol Items: Recommendations for Interventional Trials (SPIRIT) guidelines ^12^. The SPIRIT checklist is uploaded as extended data ^13^.

### Research ethics approval

This study is approved by the Institutional Ethics Committee of the Pravara Institute of Medical Sciences (Approval Number: PIMS/DR/RMC/2020/225).

### Study setting

The study will be carried out in villages close to Pravara Institute of Medical Sciences (PIMS) in Rahata taluka of Ahmednagar district in Maharashtra, India. Rahata is one of the 14 administrative blocks (taluka) in Ahmednagar district with a total population of 320,485, out of which 81.8% is rural as per the 2011 census. PIMS has a tertiary care center with a 1275 bedded multi-disciplinary facility and is located in Loni Budruk village. The antenatal clinic of the Department of Obstetrics and Gynecology provides services to approximately 150–200 pregnant women every day. This study will recruit pregnant women from four neighboring villages: Loni Khurd, Loni Budruk, Kolhar Budruk, and Kolhar-Bhagwatipur. 

### Eligibility criteria

We will first enlist all pregnant women in the study villages with the help of Anganwadi workers. We will then invite women with a gestational age between 12 weeks to 24 weeks for small group meetings in the Anganwadi centers to explain to them the proposed study. Pregnant women who are interested in participating in the study will be requested to attend the antenatal clinic of the Department of Obstetrics and Gynecology, PIMS. An eligibility assessment form will be completed by a trained Research Assistant (RA) in the antenatal clinic followed by a complete clinical examination by their obstetrician. The following inclusion/exclusion criteria will be used to assess the eligibility:

**Inclusion criteria:**

- Adult pregnant women above 18 years of age
- Gestational age between 12–24 weeks
- Planning to stay in the study area (described above) throughout study duration (approximately four months)

**Exclusion criteria:**

- Pregnant women advised rest by their obstetrician due to medical/obstetric problems
- History of two or more spontaneous abortions
- Inability to communicate in Marathi language
- Inability to attend yoga sessions
- Receiving treatment for depression or any other mental health condition
- Practicing yoga regularly for at least once a week since last four weeks

### Informed consent

A participant information sheet will be given to all eligible participants. The information sheet will include the details about the purpose of the trial, various study procedures, and contact details of the Principal Investigator and the Member Secretary of the Institutional Ethics Committee. An audio-visual version of this participant information sheet will be uploaded on the project website and the participants will be informed about how to access this. Sufficient time will be given to participants to read the information sheet and to ask any questions about the trial. They will be then invited to participate in the study. 

Eligible participants who agree to participate in the study will provide informed written consent by completing a hard copy of the consent form (see *Extended data* ^14^). Prior to signing the consent form, participants will have opportunity to review the form and ask questions about the study. Participants who are illiterate (not able to read and write Marathi, but can communicate in Marathi), will provide a verbal consent (audio-recorded) after the RA reads out the information sheet to them. An impartial third party (a family member or a health care worker) will witness the consent process and will sign the consent form.

A separate written informed consent will also be obtained at three-month follow-up assessment, for the qualitative study and for the recording (audio/video) of yoga sessions to assess fidelity and quality of intervention delivery.

RAs will be trained to ensure that the purpose of the study, randomization and other study procedures are adequately explained, and the informed consent is appropriately obtained. They will be required to sign the consent forms as well.

A copy of the participant information sheet and the informed consent form will be handed over to the participants. Those who decline to participate will be asked the reason for refusal and with their permission, their age, education, occupation, and religion will be recorded. Refusals at three-month follow-up will also be recorded.

**Additional consent provisions for collection and use of participant data and biological specimens**

The consent form will have a specific item for collection of the blood sample at the time of baseline and three-month follow-up assessment. Five milliliters of blood will be collected at each time-point to measure the level of serum C-Reactive Protein. It will ask the participants consent to share the anonymized data with other researchers and to use the anonymized data to support other research in the future.

### Interventions

**Intervention arm: Yoga-based intervention for maternal Mental health and iMmunity (Yoga-M _2_)**

Participants randomized to the intervention arm will attend weekly supervised group yoga sessions and will also be requested to practice yoga regularly at home for a total duration of three-months. Group yoga session will be for 60 to 75 minutes. A typical session will begin with an introduction of the participants, a brief overview of the session by the yoga instructor and an opening prayer. This will be followed by *sukshma-vyayam* (micro-circulation exercises) for 10 minutes, *asanas* in standing, sitting, and supine position for 15 minutes, *pranayama* for 15 minutes followed by *shavasana* (deep relaxation) for 10 minutes. The session will end with a closing prayer. A detailed description of the yoga session is provided in Table 1. After the session ends, the yoga instructor will spend time with each participant individually and suggest changes/modifications based on any problems they might have in practicing any of the yoga-based activities. Weekly group sessions will be conducted either in-person or online. In-person sessions will be preferred if allowed, depending on the COVID-19 pandemic-situation in the study area and restrictions in place. In-person sessions will be in a well aerated and ventilated hall. Use of sanitizers where appropriate and physical distancing will be maintained during yoga sessions. Participants can also attend group yoga sessions online via Zoom. The plan is to have five in-person group yoga sessions over five days in the beginning to teach the entire yoga sequence to the participants. This will be followed by weekly group yoga sessions. We anticipate that each participant will attend 10 group yoga sessions over 12 weeks.

**Table 1.** Yoga-based intervention for maternal Mental health and iMmunity (Yoga-M _2_).

	Type of Activity	Duration (Second Trimester)	Duration (Third Trimester)
**1.**	**Opening Prayer**	**3 minutes**	**3 minutes**
2.	***Sukshma-Vyayam*** **(Micro-circulation exercises)**	**10 minutes**	**5 minutes**
	*Griva Sanchalan* Neck movements	Yes	Yes
	*Skandha Paribhraman* Shoulder rotation	Yes	Yes
	*Hasta Ayama Svasanam* (Hands in and out breathing)	Yes	Yes
	*Hasta Vistara Svasanam* (Hands stretch breathing)	Yes	Yes
	*Gulpha Vistara Svasanam* (Ankle stretch breathing)	Yes	Yes
	*Karna Sanchalan* Ear movements	Yes	Yes
	*Skandha Svasanam* Shoulder movements	Yes	Yes
	*Parshva Skandha Svasanam* Shoulder cross movements	Yes	Yes
	*Hastottan Svasanam* Arms over head movements	Yes	Yes
	*Vyaghra Svasanam* (Tiger breathing)	Yes	No
	*Setu Bandha Svasanam* (Bridge posture breathing)	Yes	No
**3.**	***Asana* (Postures)**	**15 minutes**	**10 minutes**
	**Standing *Asanas***		
	*Tadasana* (Tree pose)	Yes	Yes
	*Ardhakati Chakrasan* (Lateral arc pose)	Yes	Yes
	*Trikonasan* (Triangle pose)	Yes	Yes
	**Sitting *Asanas***		
	*Vajrasana* (Ankle pose)	Yes	Yes
	*Vakrasana* (Spine twist pose)	Yes	No
	*Siddhasana* (Sage pose)	No	Yes
	*Baddhakonasana* (Bound ankle pose)	No	Yes
	*Upavistakonasana* (Spread legs pose)	No	Yes
	*Malasana* (Garland pose)	No	Yes
	**Supine *Asanas***		
	*Viparita Karani* (Half shoulder stand)	Yes	No
	*Supta-baddha Konasana* (Folded leg lumbar stretch)	Yes	Yes
**4.**	***Pranayama and* Meditation**	**10 minutes**	**20 minutes**
	*Vibhagiya Pranayam* Sectional breathing	Yes	Yes
	*Anulom-Vilom Pranayam* (Alternate nostril breathing)	Yes	Yes
	*Sheetali Pranayam* (Cooling breath)	Yes	Yes
	*Bharamari* (Humming breath)	Yes	Yes
	*Nadanusandhana* (Mind-sound resonance)	Yes	Yes
**5.**	***Dirgha Vishranti Paddhati*** **Deep Relaxation Technique**	**10 minutes**	**15 minutes**
**6.**	**Closing Prayer**	** 2 minutes**	** 2 minutes**
	**Total Duration**	**50 minutes**	**55 minutes**

In addition to the weekly supervised group sessions, participants will be encouraged to practice the yoga sequence at home daily for at least 30 minutes. The yoga instructor will work with each participant and help them develop their home practice schedule. A booklet containing a detailed description of each of the included activities and a photograph of that activity will be provided to participants. The contents of the booklet and the videos explaining the Yoga-M _2_ sequence will also be uploaded on the project website and participants will be explained to about how to access this material. A token amount will be paid to each participant in the Yoga-M _2_ arm (INR 100 per session) to cover travel expenses for attending in-person yoga sessions. Group yoga sessions will be facilitated by certified and experienced women yoga instructors. During the sessions, they will provide particular emphasis on, a) awareness of breath, b) focus on bodily sensations during each of the yoga activities, and c) avoiding injuries due to yoga practice.

In addition to the yoga practice, participants will be provided educational leaflets containing information about physical activity, sleep, and diet during pregnancy.

Yoga-M _2_ is based on the Integrated Approach for Yoga Therapy during pregnancy (IAYT-P) ^15^. This intervention was earlier evaluated using a randomized controlled trial design in an urban population in Southern India. The participants were recruited from a hospital in Bengaluru city. Women in the 20 ^th^ week of normal pregnancy were randomly assigned to receive IAYT-P group (n=51) or standard antenatal exercises (n=45). In the yoga group, there was reduction in Pregnancy related Experience (PEQ) by 26.9%, reduction in state anxiety by 15.6% and a decrease in depression by 30.7%. In the control group, there was an increase in state anxiety by 13.8% and an increase in depression by 3.6% ^15^.

Adaptations to IAYT-P were essential to include activities to improve immune function and tailor the delivery of the intervention for a rural context affected by COVID-19 crisis. This was done by reviewing the literature, undertaking a qualitative study with pregnant women and community health workers, and conducting an intervention adaptation workshop with pregnant women. Table 2 provides the summary of the intervention adaption process.

**Table 2.** Intervention adaptation process. IAYT-P: Integrated Approach for Yoga Therapy during pregnancy. MDNIY: Morarji Desai National Institute for Yoga. PIMS: Pravara Institute of Medical Sciences.

Method	Description	Changes made to IAYT-P
**Literature** ** review**	• Interventions used in previous studies (included in systematic review by Falkenberg *et.al*., ^10^) assessing the effect of yoga on immune function were reviewed in detail. • A book titled, ‘Yoga as Medicine: The Yogic Prescription for Health and Healing,’ by Dr. Timothy McCall ^16^ was referred. • Yoga manual for pregnant women developed by the MDNIY ^17^ was referred.	• Neck movements included in the manual by MDNIY were added. • Shoulder movements were added as they likely improve the flow of lymph ^16^. • Advice on diet, sleep hygiene and physical activity was added.
**Qualitative ** **Study**	• 10 in-depth interviews with pregnant women to explore facilitators and barriers to yoga practice • Three focus group discussions with community health workers to assess the feasibility of conducting group sessions in the community settings.	• Community hall or *anganwadi* center to deliver group yoga sessions instead of a hospital setup as pregnant women preferred a location close to their homes. • Modifications in a few *asanas* as some pregnant women will be able to attend the sessions wearing *saree* (a long piece of cloth draped around the body and over one shoulder, worn by women in India). • Recruitment of participants in the antenatal clinical of PIMS, as the community health workers suggested that treating obstetrician should approve the practice of yoga by pregnant women.
**Intervention ** **Adaptation ** **Workshop**	• Two intervention adaptation workshops; one with four pregnant women and other with three pregnant women were conducted to get their feedback on the modified yoga sequence.	• No changes were made in the modified sequence as all the participants provided positive feedback on the modified yoga sequence.

**Comparison arm: Enhanced Usual Care (EUC)**

Participants randomized to the EUC arm will receive a single session of health education delivered by the Intervention Coordinator (IC). During this session, aspects related to sleep hygiene and diet during pregnancy will be discussed and they will be encouraged to undertake regular physical activity (150 min/week). They will be provided educational leaflets containing information about physical activity, sleep, and diet during pregnancy. The IC will work with the participants to identify ways to reduce stress during pregnancy and improve social support. As per the current standard of care in the antenatal clinic of Department of Obstetrics, pregnant women are neither screened for stress nor is there any discussion about improving mental health during pregnancy. Participants with moderate stress (Perceived Stress Scale score > 13) will be referred to a psychiatrist in PIMS.

**Criteria for discontinuing or modifying allocated interventions**

If there is spontaneous abortion or stillbirth, participation in both the arms will be discontinued. Other than this, there is no specific criteria to discontinue Yoga-M _2_ intervention. A few modifications in the sequence for individual participants may be done by the yoga instructor based on feedback from them. Obstetrician may advise participants to discontinue yoga due to health issues or they may decide to stop practicing yoga themselves for any reason.

**Strategies to improve adherence to interventions**

The IC and the yoga instructor will regularly contact participants in Yoga-M _2_ arm to coordinate attendance in weekly group yoga sessions. If a participant misses two consecutive weekly group yoga sessions, the IC will visit the participant at home to address any barriers related to joining the session. Adherence to home practice will be assessed using home practice logs. 

**Relevant concomitant care permitted or prohibited during the trial**

Throughout the duration of the trial, participants will receive usual care and no specific component of antenatal care or other health services will be prohibited.

**Provisions for post-trial care**

Participants in both arms will receive information about yoga classes they can join after the end of the trial. Participants in the control arm will also receive the yoga intervention booklet and the audio-visual material. Participation in yoga classes post-trial will be entirely voluntary and on a self-pay basis. No compensation will be provided to participants who suffer non-negligent harm from trial participation.

**Outcomes**

Outcome assessment will include measures related to:

a) feasibility and acceptability of yoga-based intervention (Yoga-M _2_) delivery
b) preliminary efficacy of Yoga-M _2_

Feasibility and acceptability will be assessed using the following process data:

1. Number and proportion of eligible participants who are recruited per week in each arm.
2. Number and proportion of recruited participants in Yoga-M _2_ arm who attend the in-person yoga sessions.
3. Number and proportion of recruited participants in Yoga-M _2_ arm who attend at least 50% of the follow-up yoga sessions.
4. Number and proportion of recruited participants who complete follow-up assessment three-months post-randomization in each arm.

Process data will be collected from the study enrollment logs and intervention delivery logs. Study enrollment logs will be maintained by RAs and intervention delivery logs will be maintained by the IC. Every week this data will be manually entered and stored as CSV files on an access-restricted computer.

Assessment of acceptability of the intervention will also be done using a structured satisfaction survey and a qualitative study. In-Depth Interviews (IDIs) with all yoga instructors and enrolled participants will be completed. Semi-structured interviews will cover themes related to the delivery, content, comfort, and complexity of the intervention. We plan to enroll around 15 participants (10 from the intervention arm and 5 from the control arm), however, the sample size for the qualitative study will be determined by data saturation.

The description of measures which will be used to assess the preliminary efficacy of the intervention is given in Table 3.

**Table 3.** Measures to assess the preliminary efficacy.

Outcome	Measure	Time-point
Stress	Perceived Stress Scale (PSS)	Baseline and three-months post-randomization
Quality of Life	EuroQoL 5 Dimensions Score (EQ-5D-5L)	Baseline and three-months post-randomization
Immune Function	Wisconsin Upper Respiratory Symptom Severity Scale	Baseline and three-months post-randomization
Immune Function	Serum C-Reactive Protein	Baseline and three-months post-randomization
Injuries due to yoga	Self-report log	Every week for three-months post-randomization
Serious Adverse Events (SAE)	SAE report form	Baseline and three-months post-randomization

**Perceived Stress Scale (PSS)**

Perceived stress will be measured using 10-item version of the original scale ^18^. The items in this instrument assess how unpredictable, uncontrollable, and overloaded participants appraise the situations in their lives in last one month. PSS has been widely used in research studies across the globe and it has acceptable psychometric properties ^19^. In this study, we will use the Marathi version of the PSS which was translated from the English version using the World Health Organization (WHO) guidelines for the process of translation and adaption of instruments ^20^.

**EuroQoL 5 Dimensions Score (EQ-5D-5L)**

European Quality of Life Five Dimension (EQ-5D-5L) descriptive system will be used to measure Health related Quality of Life (HRQoL). A single utility score will be calculated using the value set/preference weights for Thailand as the Eq-5D value set for India is under development ^21^. Participants will also provide an overall evaluation of their health using a visual analogue scale (VAS). We will use the Marathi version of EQ-5D-5L with permission from the EuroQoL Group ^22^. The EQ-5D has been validated in different parts of the world ^23^. 

**Wisconsin Upper Respiratory Symptom Severity Scale (WURSS)**

The incidence and severity of upper respiratory tract infections in the participants will be assessed using the Wisconsin Upper Respiratory Symptom Survey (WURSS-21). This is a 21-item scale; 10 items assess symptoms, nine items assess functional impairments, and there is one item each to assess the overall severity and change. Responsiveness, reliability, convergence, and importance to patients of this scale have been validated ^24^. Participants will complete WURSS-21 at the end of each day in the study. We will use the Marathi version of WURSS with permission from the developers.

**Serum C-Reactive Protein (CRP)**

CRP has been proposed as an objective marker to measure wellness ^25^. CRP is a nonspecific maker of inflammatory process, the serum levels increase with body insult, tissue damage, aging, and cardiovascular diseases. Bacterial (or viral, fungal) infections lead to a marked increase in serum CRP levels. Although it is not a diagnostic marker, it can be used to track the inflammatory process, overall wellness, and individual’s quality of life. Lifestyle changes such as cessation of smoking, increasing physical activity and reducing body mass index led to a decrease in serum CRP levels ^25^. Yoga and other mind-body techniques reduce inflammation through a reduction in CRP ^26^. An eight week Hatha yoga program compared to standard medical treatment was found to significantly reduce serum concentrations of interleukin 6 and CRP in two randomized controlled trials ^27, 28^. Serum CRP levels will be determined using fixed-point immune-rate method (VITROS 250/350/5, 1 FS/4600/XT 3400 Chemistry Systems and the VITROS 5600/XT 7600 Integrated Systems). The limit of detection for VITROS Chemistry Products CRP slides is 2.72 mg/L.

**Self-report log**

Participants in both arms will maintain a log to document any injuries/non-serious adverse events during the duration of the trial.

### Participant timeline

**Figure 1.**
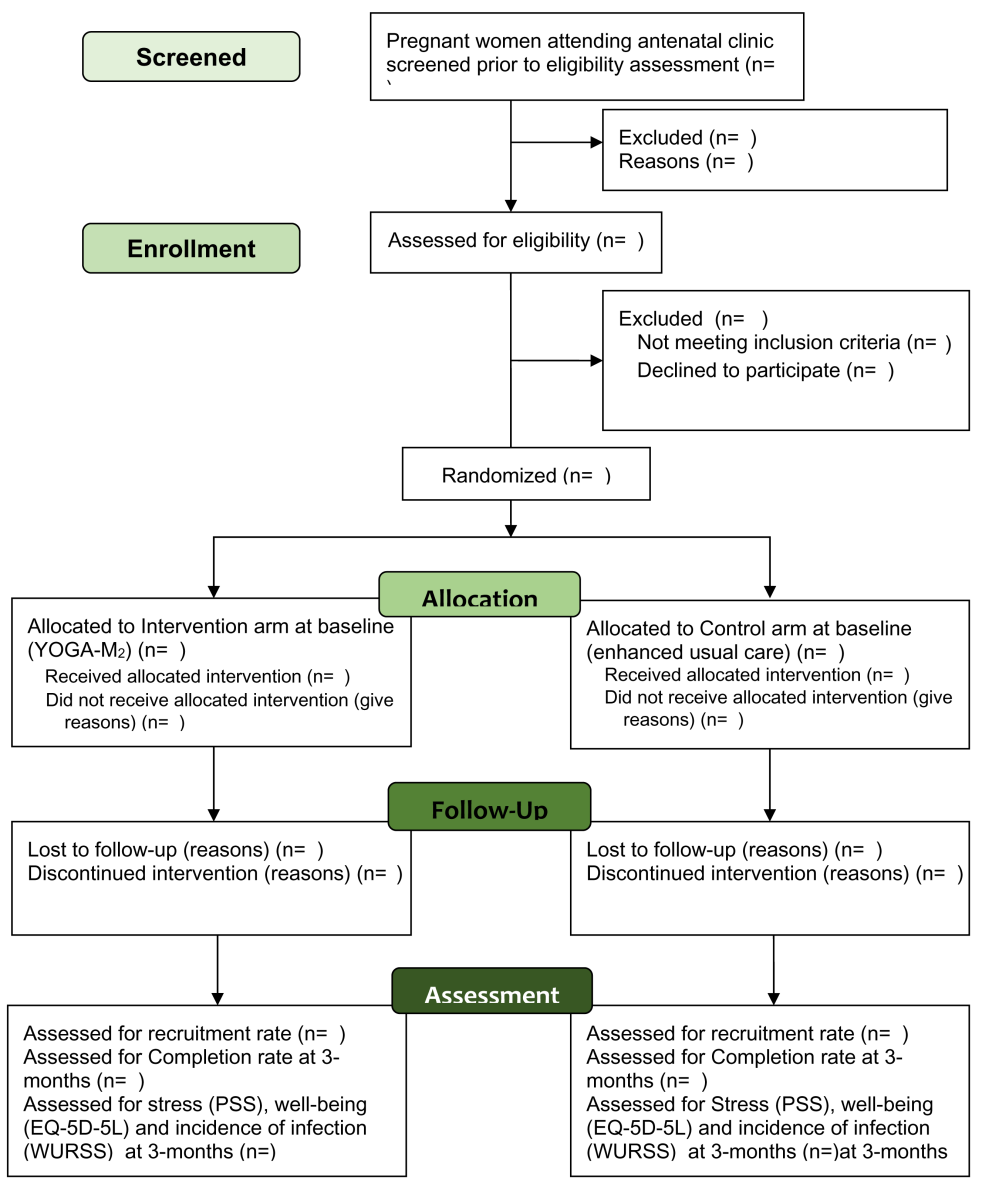
Participant flow diagram through the pilot randomized trial.

Participant’s timeline through the trial is presented in Figure 1.

### Sample size

We anticipate that the standardized effect size between the two arms will be small (0.2). Hence, we plan to recruit 25 pregnant women in each arm (total=50) based on the recommendations by Whitehead *et al.* ^29^.

### Recruitment

Participants will complete baseline assessment using a questionnaire after providing the informed consent. RAs will administer this questionnaire and it will include data on demographic and socio-economic measures and the primary and secondary outcome measures. The IC will then undertake treatment allocation in a separate room. The IC will work independently of RAs who will not be aware about the treatment allocation.

### Assignment of interventions: allocation

**Sequence generation**

An independent statistician will generate randomization list using the statistical program R (version 3.6.1). First, a list of variable block sizes (randomly selected block sizes of 2/4/6/8) will be created followed by a list of intervention allocation (Yoga-M _2_ or EUC) using a 1:1 allocation ratio. The list will be shared with the Clinical Research Coordinator (CRC) based in the Directorate of Research, PIMS. They will prepare serially numbered, opaque, sealed envelopes using the randomization list.

**Concealment mechanism**

Participants will be randomly allocated to the intervention group by the IC using serially numbered, opaque sealed envelopes after they complete their baseline assessment.

**Implementation**

The IC will collect a set of opaque sealed envelopes from CRC as per the serial numbers, a few hours prior to the recruitment. The IC will open the opaque sealed envelopes (as per the serial numbers) in front of the participants and immediately inform them about the intervention group (i.e., Yoga-M _2_ or EUC) they have been assigned to. Participants will be requested to not reveal their group assignment to the RAs at the three-month follow-up assessment. To maintain allocation concealment, neither the RAs nor any staff working at the hospital will have access to the randomization lists. The IC will inform the CRC the unique identification number of the participant (and no other details) and the intervention allocation group. The CRC will maintain the record of intervention allocation.

### Assignment of interventions: blinding

**Who will be blinded?**

Participants, their family members, and study team members involved with intervention delivery (IC and yoga instructors) will be aware of participants’ assigned intervention during the trial. It is not possible to avoid bias arising due to this. RAs will complete baseline and three-month follow-up assessments, and they will not interact in any way with the intervention team. RAs will not be aware of the intervention allocation status. Participants and family members will be instructed not to disclose whether they are receiving the intervention to the RAs at the time of three-month follow-up assessment. Additionally, RAs will also remind participants not to reveal their group assignment to them so that they remain blind to the treatment allocation of participants.

Obstetricians will not be aware of the intervention allocation status, minimizing the risk of any change in their behavior while dealing with the participants.

The intervention team led by the IC and the outcome assessment team comprising of RAs will not have any interactions during the trial. They will work independently of each other in separate locations and will have no communication about trial participants and their intervention allocation status. During the training of RAs in the outcome assessment team, special emphasis will be given to explaining the equipoise between the two interventions.

We will monitor the risk of contamination at the level of the yoga instructors, intervention coordinator, and participants. Yoga instructors and the intervention coordinator will be requested to not share the information about Yoga-M _2_ with pregnant women other than those enrolled in the intervention arm (Yoga-M _2_). They will also ensure that participants in both the arms adhere to the group randomly allocated to them.

**Procedure for unblinding if needed**

In case there is any yoga-related adverse event, allocation of intervention group will be revealed to obstetrician.

### Data collection and management

**Plans for assessment and collection of outcomes**

Case Record Forms (CRF) will be used by RAs to collect data at baseline and at three-months follow-up assessment. Both the baseline and follow-up assessment will be completed in the antenatal clinic of the Pravara Institute of Medical Sciences. Pen and paper format will be used for data collection. Data will be converted into digital format by manual entry using Microsoft Excel. Data will be stored as a CSV (comma-separated values) file. There will be a date and time stamp of the original data entry and any subsequent change to the document will be noted in an audit trail. Data related to the fidelity and quality of yoga intervention delivery will also be in paper form and similar process will be used to enter and store data as a CSV file. Digital voice recorders will be used to collect qualitative data. A document containing memos and field notes will be maintained. The audio data will be transcribed in Marathi and then translated in English for analysis. The data will be anonymized but linked with the trial ID.

For each data source, the CRC will perform the data, range, and consistency checks at weekly intervals. The CRC will resolve the queries promptly and will update the database and maintain the audit trail. Initially, separate databases will be used to store different types of data. Once the data collection is complete, each individual database will be locked, and these separate databases will be merged into a master. Passwords will be used by the CRC to protect the data. No member of the trial team will have access to these passwords.

**Plans to promote participant retention and complete follow-up**

At the baseline, participants will be informed about the three-month follow-up assessment and an interview will be scheduled coinciding with their antenatal check-up. A reminder phone call will be made one week prior to the follow-up assessment. A seven-day window period after the due date will be allowed to complete the assessment. In case the participants are unable to visit the antenatal clinic, a home-visit will be planned as per the convenience of the participant.

**Data management**

A Trial Master File containing all the essential documents will be maintained. Trial documentation will be maintained for seven years after the end of the trial. At the end of this period, data in paper format will be securely disposed and data in electronic format will not contain any identifiable information.

All CRFs and other documents related to the study will be stored securely in the Directorate of Research, PIMS. Password-protected servers will be used to store all electronic records. To ensure participant confidentiality, coded identification numbers will be used to identify participant data. Participant information in paper format will be securely stored in locked cabinets. Anonymized data will be used for analysis and for presenting results at conferences and in scientific journals.

**Confidentiality**

The identity and privacy of research participants will be protected. Any variable which may help to infer the identity of any participant, will be deleted from the database.

### Statistical methods

Statistical analyses will be conducted in Stata version 14.2 by the trial statistician (ST). Descriptive statistics (means and standard deviations or proportions) of sociodemographic characteristics and outcome measures will be provided for all trial participants at baseline and three-months after enrollment; this will include means and standard deviations, or proportions, in the two arms, as appropriate. The primary analysis will be intention-to-treat. Binomial regression models will be used to estimate risk ratios and 95% confidence intervals for binary outcomes, while linear regression models will be used to estimate regression coefficients for continuous outcomes. Analyses of continuous outcomes will additionally adjust for the baseline value of that outcome and any imbalances of baseline values of key covariates.

**Methods for additional analyses (e.g., subgroup analyses)**

***Process evaluation analysis.*** IDIs and Focus Group Discussions will be audio-recorded, and field notes will be taken. English interviews will be transcribed verbatim while the interviews conducted in Marathi will be transcribed and translated into English. Bilingual team members, fluent in English and Marathi will carry out back-translation checks. Qualitative data will be analyzed using framework analysis approach. An *a priori* coding framework with a set of high-level themes will be developed. Lower order themes will be inductively derived from the data. NVivo 9 software (QSR) will be used to code the data. Principal investigator will be primarily responsible for the data analysis and synthesis of the findings.

**Methods in analysis to handle protocol non-adherence and any statistical methods to handle missing data**

Complier Average Causal Effect (CACE) analysis will be carried out to assess the effect of receiving treatment as defined in the protocol. This will be done in three ways. Full compliance is defined as attendance at three or more of the first five sessions and at least four other sessions. The first type of analysis will involve fully compliant participants. In the second type of analysis, participants with any compliance (attendance at one yoga session or more) will be included. The third type of analysis will include the number of sessions attended in its continuous form. The extent and pattern of missing data for each outcome will be explored. Multiple imputation of missing values will be performed if there are more than 10% missing values at endpoint.

**Plans to give access to the full protocol, participant level data and statistical code**

The full protocol will be available on a dedicated website: 

https://rahulshidhayelab.in/yogammmc/

### Oversight and monitoring

**Composition of the coordinating centre and trial steering committee**

The Trial Management Group (TMG) will coordinate the day-to-day implementation of the trial. The Trial Steering Committee (TSC) will provide the oversight for the trial. TMG will comprise of the principal investigator, the RAs, the IC, ST, a clinician, and a patient representative. It will meet every two weeks. The TSC will meet every four weeks. It will be headed by an independent chairperson and there will be four other members. There will be representatives of the TMG on the TSC.

**Composition of the data monitoring committee, its role and reporting structure**

Due to the nature of the trial and low risk for participants, the TSC will also take on the role of the data monitoring committee. During the TSC meetings, data related to progress of the trial and safety of participants will be reviewed and discussed.

**Adverse event reporting and harms**

Previous evidence suggests that yoga during pregnancy is safe ^30^ and no adverse events have been reported in an earlier trial by Satyapriya *et al.* ^15^. Records of all Serious Adverse Events (SAEs) will be maintained in a log by the CRC.

1) Death due to any cause
2) Hospitalization due to any cause
3) Suicide attempt
4) Spontaneous abortion or stillbirth

SAEs in both arms will be documented by the RAs at three-month follow-up assessment. The IC and yoga instructors will also be trained to identify SAEs and notify them to the CRC. Within two weeks of becoming aware of any SAE (related or unexpected), the Institutional Ethics Committee will be informed about the same by PI. A standard operating procedure will include all the details pertaining to the detection, appropriate response and reporting of SAEs. If any SAE requires an immediate response, an independent medical specialist will do so within two working days. The criteria for unblinding of adverse events will be set *a priori*. A summary of all adverse events will be sent to the Institutional Ethics Committee, the funder, and the TSC regularly as part of the progress report.

All necessary and appropriate medical assistance will be provided to participants in the PIMS for injuries related to participation in the trial (intervention arm). There will be no financial burden on the participants for trial related injuries and adverse events.

**Frequency and plans for auditing trial conduct**

The sponsor and the Institutional Ethics Committee will monitor the aspects of the study on an ongoing basis.

**Plans for communicating important protocol amendments to relevant parties (e.g., trial participants, ethical committees)**

Approval from the TSC and the sponsor will be obtained for any modifications in the protocol that will affect the scientific aspects of the study, participant safety, scientific value of the study, or the way in which study is conducted. These modifications will be submitted to the Institutional Ethics Committee as amendments to the protocol. Changes made to the protocol will be communicated to the trial participants through written communication. The Final report submitted to the funder will enlist all the amendments to protocol.

### Sponsor information

Name and contact information of the trial sponsor: Pravara Institute of Medical Sciences (PIMS; ethicssupport@pmtpims.org)

The sponsor did not have any role in designing the trial. It will also not have any role in data collection, data management, analysis as well as interpretation of data. The sponsor will not have any part in writing the report and decisions related to submission of the report for publication.

### Dissemination plans

Publications in open-access peer-reviewed journals will be the dominant mechanism for communicating the key findings of our research. The final peer-reviewed journal manuscripts that arise from this research will be archived in PubMed Central (or a similar digital archive) once they are accepted for publication. Dr. Shidhaye and other members of the study team will present research data at national and international scientific conferences (including oral presentations, posters, and published abstracts) prior to publication of the findings. Study reports with interpretation of results and recommendations will be presented to all major stakeholders. Results of the study will be presented to all participating communities through leaflets, flyers, local media, and a series of interactive meetings with the community members.

Dataset will be available on request two years after the completion of the trial

## Discussion

This pilot randomized controlled trial will generate evidence on the feasibility, acceptability, and preliminary efficacy of a yoga-based intervention to improve maternal mental health and immune function during the ongoing COVID-19 crisis. To the best of our knowledge there is only one study to date which has assessed the effect of yoga on immune function during pregnancy. Many randomized controlled trials have studied the effect of yoga on depression and anxiety during pregnancy, but this study is probably the first from a low-resource rural setting in India.

Generally, yoga during pregnancy is safe, low-cost, can be easily incorporated as part of ‘self-care’ routine, has beneficial effect on multiple body functions, and is relatively easy to scale due to its cultural acceptability in the Indian context. However, we anticipate a few challenges in implementation of this trial.

The first and foremost challenge is posed by the current COVID-19 crisis. At present there are few active COVID-19 cases in the study area but given the extent of social interaction and the rise of infections due to COVID-19 variants (e.g., omicron), a possibility of the next wave and subsequent lock-down and restrictions on all activities cannot be completely denied. If this happens, it will be difficult to recruit the participants in the trial. To mitigate this challenge, we decided to restrict the inclusion of participants only from four nearby villages and provide an option of having group yoga sessions conducted on Zoom.

The second formidable challenge relates to lack of adherence to the yoga protocol as participants in the active intervention arm may not be too motivated to practice yoga on a regular basis and attend weekly group sessions. We conducted in-depth interviews with pregnant women in the study area to explore this further and involved them in the process of adaptation of the intervention. We also actively engaged with the community health workers in the study area and got their inputs on the delivery of the intervention by undertaking focus group discussions (Shidhaye, unpublished data). All participants will be assessed by their treating obstetrician who will not only sign-off on the participation in the study but will also encourage the participants to maintain adherence. This will be done for all the participants and will take place before randomization.

Transportation is the third important challenge which we will try to address by timing the recruitment, baseline, and follow-up assessment of participants with their routine ante-natal care visits. In case they need to come to antenatal clinic for study related activities, we will provide them travel allowance for the same. Group yoga sessions will be conducted in *Anganwadi* centers or a community hall, closer to the homes of the participants. This was suggested by the participants in in-depth interviews.

We also anticipate that ‘clothing’ may pose a challenge to undertake all the activities included in the intervention. Many pregnant women in rural areas are not comfortable in wearing anything other than ‘ *saree*’. This will require adaptation in some *asanas* as described earlier.

These and any other challenges will be discussed with yoga experts and stakeholders throughout the course of the trial and appropriate modifications will be made to the intervention.

One of the major limitations of this trial is our inability to assess the efficacy of the intervention on pro-inflammatory markers (other than serum CRP), cortisol (hair and salivary) and serum immunoglobulin levels. These assessments are not done in PIMS and the costs for undertaking these assessments from outside laboratories is beyond the scope of funding for this study.

The expected outcome of this trial is a structured intervention manual, finalization of the evaluation strategy and a protocol to undertake a larger randomized controlled trial. If we find that it is feasible to deliver Yoga-M _2 _and that it is acceptable to the participants, we will apply for further research funding to conduct an explanatory randomized controlled trial to assess the efficacy and cost-effectiveness of Yoga-M _2_ on immune function in pregnant women. 

We are also hopeful that the findings of this trial will inform the plan of the Government of India to integrate yoga with primary health care through, Health and Wellness Centre, established as part the of the ‘ *Ayushman Bharat Yojana*,’ or the National Heath Protection Scheme. To this effect, we will make all efforts to communicate the findings of this study with senior health officials in the district and state administration.

## Data availability

### Underlying data

No data is associated with this article

### Extended data

Figshare: Participant Information Sheet and Informed Consent forms. https://doi.org/10.6084/m9.figshare.19306757.v1 ^13^.

Data are available under the terms of the Creative Commons Zero "No rights reserved" data waiver (CC0 1.0 Public domain dedication).

### Reporting guidelines

Figshare: SPIRIT checklist for ‘Yoga to improve maternal mental health and immune function during the COVID-19 crisis: study protocol for a pilot randomized controlled trial’. https://doi.org/10.6084/m9.figshare.19306784.v2 ^14^.

Data are available under the terms of the Creative Commons Zero "No rights reserved" data waiver (CC0 1.0 Public domain dedication).
