# Supplementary material for: Yoga to improve maternal mental health and immune function during the COVID-19 crisis (Yoga-M 2 trial): study protocol for a pilot randomized controlled trial
Source: Wellcome Open Res. 2022 Aug 4;7:109. Originally published 2022 Mar 23. [Version 2] doi: 10.12688/wellcomeopenres.17729.2 (PMC9360909; doi:10.12688/wellcomeopenres.17729.2)
Supplement: Peer review history

# Peer review: Reviewer response for version 2

**DOI:** 10.21956/wellcomeopenres.20039.r51864

**Published:** 2022-08-08

**Authors:** Melissa O'Shea

**Affiliations:** 1 School of Psychology, Deakin University, Geelong, VIC, Australia

I thank the authors for their attention to the feedback and considerations I provided in my initial review. Assuming that issues that were agreed to be amended but have yet to be incorporated into the revised manuscript are addressed, I welcome the approval of this protocol.

Is the study design appropriate for the research question?

Yes

Is the rationale for, and objectives of, the study clearly described?

Partly

Are sufficient details of the methods provided to allow replication by others?

Yes

Are the datasets clearly presented in a useable and accessible format?

Not applicable

Reviewer Expertise:

Integrative Psychology

I confirm that I have read this submission and believe that I have an appropriate level of expertise to confirm that it is of an acceptable scientific standard.

# Peer review: Reviewer response for version 2

**DOI:** 10.21956/wellcomeopenres.20039.r51863

**Published:** 2022-08-04

**Authors:** Carolina Estevao

**Affiliations:** 1 Kings College London, London, UK

The authors have addressed my comments appropriately and I believe this manuscript is now ready for publication. I look forward to seeing the results of this very relevant study.

Is the study design appropriate for the research question?

Yes

Is the rationale for, and objectives of, the study clearly described?

Yes

Are sufficient details of the methods provided to allow replication by others?

Yes

Are the datasets clearly presented in a useable and accessible format?

Not applicable

Reviewer Expertise:

Perinatal mental health, community-based interventions, low-income population research, yoga, psychiatry, psychology

I confirm that I have read this submission and believe that I have an appropriate level of expertise to confirm that it is of an acceptable scientific standard.

# Peer review: Reviewer response for version 1

**DOI:** 10.21956/wellcomeopenres.19619.r51081

**Published:** 2022-07-18

**Authors:** Melissa O'Shea

**Affiliations:** 1 School of Psychology, Deakin University, Geelong, VIC, Australia

I commend the research team for their efforts to expand ante-natal health care treatments for rurally located women in India, particularly after their healthcare services have been further eroded during the COVID 19 pandemic. As the researchers describe, yoga offers benefits to women during pregnancy, including for their wellbeing and risk of stress and respiratory related illness. The proposed trial of a yoga-based intervention against Enhanced usual care ( usual care with a single session of health education ) is generally well considered and robust. I wish the research group well in progressing the trial.

 If I can make a couple of suggestions to strengthen their publication:

 I encourage the group to temper language that relates to the mental health risks for women during pregnancy; pregnancy can also enhance many women's mental health. They might like to contrast rates of Depression during other stages of life. This is not to say, when depression occurs during this stage of life, that it is not worthy of discrete attention. It is also not to say that yoga is a potentially helpful intervention for all women during this period. This rationale could be built more strongly - ie the potential benefits of yoga for pregnant women, in reducing risks for mental health disorders inc depression over the period (and thus also, complications of this for the baby), enhancing wellbeing and reducing stress, as well as building immunity. On this final point, whilst COVID 19 is likely to be relevant, it is perhaps overemphasised in this study. I would also like to see the rationale for this particular study built a little more. The gaps in the literature could be more clearly described upfront, including a discussion of the existing study of Yoga-M2 and how this trial will build on the findings from it.

 Regarding the method, once again, just a couple of suggestions:

**Exclusions:**

- Having inability to attend: will the reasons for inability to attend be analysed in some way as this also goes to the feasibility of yoga intervention?
- Also, consider 'miscarriage' rather than abortion for the wider audience.
- Receiving treatment for depression of other mental health conditions (just noting that this does not leave open the opportunity to see how yoga might complement these other therapies, but perhaps the next study!).

**Outcomes:**

- Just a query as to whether completion of a 3-month follow-up goes to acceptability or feasibility?
- The researchers might like to consider whether self administration of the Respiratory symptom severity scale everyday is too onerous?
- Could there be a brief discussion regarding the link between immunity and the Serum C-reactive protein for the naive reader?
- Qualitative analysis methods could be elaborated; the researchers might also consider the use of template thematic analysis

**Yoga Intervention:**

- Well described and relevant to a population of pregnant women (focus on gentle asana, breath work and allowing for modifications for safety and stage of pregnancy).

**Discussion:**

- The discussion of potential challenges to the successful implementation of the trial is helpful, as is intended outcomes.  

Is the study design appropriate for the research question?

Yes

Is the rationale for, and objectives of, the study clearly described?

Partly

Are sufficient details of the methods provided to allow replication by others?

Yes

Are the datasets clearly presented in a useable and accessible format?

Not applicable

Reviewer Expertise:

Integrative Psychology

I confirm that I have read this submission and believe that I have an appropriate level of expertise to confirm that it is of an acceptable scientific standard, however I have significant reservations, as outlined above.

# Response

**Published:** 2022-08-04

**Authors:** Rahul Shidhaye

**Affiliations:** Pravara Institute of Medical Sciences , India

Dear Dr. O’Shea,

**Re: Manuscript title: Yoga to improve maternal mental health and immune function during the COVID-19 crisis (Yoga-M2 trial): study protocol for a pilot randomized controlled trial.**

 Many thanks for taking out time to review our submission and providing very valuable comments and suggestions. We have responded to each of your comments in italics below and believe we have been able to address all the comments. We have made corresponding changes to the manuscript and the revised manuscript is uploaded.

Comment # 1:

 I commend the research team for their efforts to expand ante-natal health care treatments for rurally located women in India, particularly after their healthcare services have been further eroded during the COVID 19 pandemic. As the researchers describe, yoga offers benefits to women during pregnancy, including for their wellbeing and risk of stress and respiratory related illness. The proposed trial of a yoga-based intervention against Enhanced usual care (usual care with a single session of health education) is generally well considered and robust. I wish the research group well in progressing the trial.

Response:

*Thank you so much for your positive response and kind wishes for the implementation of the pilot trial.*

Comment # 2:

 I encourage the group to temper language that relates to the mental health risks for women during pregnancy; pregnancy can also enhance many women's mental health. They might like to contrast rates of Depression during other stages of life. This is not to say, when depression occurs during this stage of life, that it is not worthy of discrete attention. It is also not to say that yoga is a potentially helpful intervention for all women during this period. This rationale could be built more strongly - ie the potential benefits of yoga for pregnant women, in reducing risks for mental health disorders inc depression over the period (and thus also, complications of this for the baby), enhancing wellbeing and reducing stress, as well as building immunity. On this final point, whilst COVID 19 is likely to be relevant, it is perhaps overemphasised in this study. I would also like to see the rationale for this particular study built a little more. The gaps in the literature could be more clearly described upfront, including a discussion of the existing study of Yoga-M2 and how this trial will build on the findings from it.

Response:

*We thank the reviewer for this suggestion. It is indeed important to discuss the positive aspects of pregnancy on woman’s mental health. We have modified the background section and have added the following sentences in the beginning,*

*‘The experience of pregnancy can be both exciting and challenging for a woman and her family. Pregnancy is associated with happiness* *(Malhotra et al. 2015)* *and increase in mental aspect of quality of life* *(Lagadec et al. 2018)* *.’*

*We also completely agree that COVID-19 has been overemphasized in the introduction. There are two reasons for this. First, the Delta-Omicron wave of COVID-19 was quite active in India and other parts of the globe at the time of the submission of the manuscript. Second, this work is funded by the Department of Science and Technology, Government of India with a specific purpose of identifying* *appropriate yoga and meditation-based interventions to fight COVID-19 and other similar kind of viruses. The study protocol of this trial is in alignment with the grant proposal which the funder had approved, hence, the importance of developing a yoga-based intervention to improve immune function and well-being during pregnancy in the context of COVID-19 is included in the rationale.*

*We do agree with the reviewer’s suggestion and have modified the background section which is appended below. To strengthen the rationale of the study, we now explain in detail the relationship between yoga, infection, and the markers of inflammatory process (C-Reactive Protein) in this section.*

***‘Background***

*The experience of pregnancy can be both exciting and challenging for a woman and her family. Pregnancy is associated with happiness* *(1)* *and increase in mental aspect of quality of life* *(2)* *. However, it can also be a period of increased physical and psychological vulnerability.* *Changes in cardiorespiratory and immune system functioning increase the susceptibility of women to severe infection and hypoxic compromise* *(3)* *. This is especially important during the ongoing COVID-19 crisis as COVID-19 during pregnancy can increase the risk of preeclampsia, venous thromboembolism, premature fetal membrane rupture, or preterm delivery* *(4,5)* *. Women living in social adversity can experience mental health conditions, especially depression which is quite common during this period. One in four women in South Asia is likely to have depression during pregnancy, while in India, the pooled prevalence of antenatal depression is 17.7% (95% CI: 11.2%–26.9%)* *(6)* *. Antenatal depression is associated with higher risk of pregnancy-related mortality and morbidity, pre-term delivery, low birth-weight, post-partum depression and adverse neuro-cognitive developmental outcomes in infants, children and adolescents* *(7)* *.*

*Yoga is a mind-body practice, and its roots can be traced in ancient Indian philosophy. Yoga has been practiced in India for more than 7000 years* *(8)* *. Yoga during pregnancy can be very helpful in prevention and treatment of stress and depression* *(9)* *. A meta-analysis found a positive effect of yoga on antenatal depression* *(10)* *. Another systematic review assessing the effect of yoga on immune system functioning found that yoga can down regulate pro-inflammatory markers and enhance cell-mediated and mucosal immunity. This makes yoga a suitable intervention for individuals suffering from diseases with inflammatory component and those who are at higher risk of getting infections* *(11)* *. There is only one study which has assessed the effect of yoga on immune function during pregnancy* *(12)* *. In this study, yoga-based intervention, which included postures, deep breathing, guided imagery, and deep relaxation was delivered to 48 women during pregnancy, 70 minutes per session, 2 sessions per week for 20 weeks. Pregnant women who practiced yoga had higher immunoglobulin A levels and lower salivary cortisol levels immediately after a yoga session, compared to those who did not practice yoga. In the long-term, salivary immunoglobulin A levels were significantly higher in the yoga group compared to the control group* *(12)* *.*

*How to assess the effect of yoga on inflammatory process is a key question. C-Reactive Protein (CRP) is a nonspecific maker of inflammatory process, the serum levels increase with body insult, tissue damage, aging, and cardiovascular diseases. Bacterial (or viral, fungal) infections lead to a marked increase in serum CRP levels. Although it is not a diagnostic marker, it can be used to track the inflammatory process, overall wellness, and individual’s quality of life. Lifestyle changes such as cessation of smoking, increasing physical activity, reducing body mass index led to a decrease in serum CRP levels* *(13)* *. Yoga and other mind-body techniques reduce inflammation through a reduction in CRP* *(14)* *. An eight week Hatha yoga program compared to standard medical treatment was found to significantly reduce serum concentrations of interleukin 6 and CRP in two randomized controlled trials* *(15,16)* *.*

***Rationale for study***

*The possibility of another COVID-19 wave in India (and other parts of the globe) can’t be entirely ruled out. This necessitates generating evidence for population-based interventions which can improve immune system functioning and reduce the susceptibility of infections in vulnerable groups like pregnant women. Yoga can be practiced by pregnant women as self-care with minimal training. Yoga can play an important role in reducing stress and improving well-being of pregnant women. However, there are no studies from India which have assessed the effects of yoga-based intervention on immune system functioning and mental health of pregnant women.’*

Comment # 3:

 Exclusions:

 Having inability to attend: will the reasons for inability to attend be analysed in some way as this also goes to the feasibility of yoga intervention?

 Also, consider 'miscarriage' rather than abortion for the wider audience.

 Receiving treatment for depression of other mental health conditions (just noting that this does not leave open the opportunity to see how yoga might complement these other therapies, but perhaps the next study!).

Response:

*Thanks for all these important suggestions related to exclusion criteria.*

*We completely agree with the reviewer that, ‘inability to attend yoga sessions’, points towards less feasibility of this intervention. We will certainly document and analyse the reasons for exclusion of participants and for those pregnant women who mention, ‘inability to attend yoga sessions’, we will specifically ask them about why it would be difficult for them to attend.*

*We have now included the term, ‘miscarriage’, in the exclusion criteria.*

*The revised text is as follows,*

*‘History of two or more miscarriages (spontaneous abortions)’*

*We also agree that excluding women who are receiving treatment for depression, or any other mental condition would prevent us from assessing the impact of how yoga can complement the existing treatment. But if we include pregnant women who are receiving treatment for depression, then we may not be able to establish if the change in perceived stress score or quality of life is due to ongoing treatment for depression or due to Yoga-based intervention. Hence, we decided to exclude women currently receiving treatment for depression. As the reviewer has suggested we will re-think about this criterion if we get an opportunity in future to undertake definitive/explanatory trial.*

Comment # 4:

 Outcomes:

 Just a query as to whether completion of a 3-month follow-up goes to acceptability or feasibility?

Response:

*We plan to report the number and proportion of recruited participants who complete follow-up assessment three-months post-randomization in both the Yoga-M _2_ arm and the Enhanced Usual Care arm. Thus, this is primarily a measure of feasibility for outcome assessment.*

*The measures for acceptability of Yoga-M _2_ intervention will be, *

- *Number and proportion of recruited participants in Yoga-M _2_ arm who attend the in-person yoga sessions.*
- *Number and proportion of recruited participants in Yoga-M _2_ arm who attend at least 50% of the follow-up yoga sessions.*

*Acceptability of the intervention as well as feasibility to attend these sessions will also be assessed by conducting in-depth interviews with trial participants in Yoga-M _2_ arm.*

Comment # 5:

 The researchers might like to consider whether self-administration of the Respiratory symptom severity scale everyday is too onerous?

Response:

*We do feel that completing the Wisconsin Upper Respiratory Symptom Severity Scale is burdensome for participants. Exploring the feasibility of outcome assessment is also one of the objectives of this trial. We do wish to explore this as part of the qualitative study with trial participants and will report the findings in main trial paper later.*

Comment # 6:

 Could there be a brief discussion regarding the link between immunity and the Serum C-reactive protein for the naive reader?

Response:

*We have now included this in the background section as follows,*

*‘How to assess the effect of yoga on inflammatory process is a key question. C-Reactive Protein (CRP) is a nonspecific maker of inflammatory process, the serum levels increase with body insult, tissue damage, aging, and cardiovascular diseases. Bacterial (or viral, fungal) infections lead to a marked increase in serum CRP levels. Although it is not a diagnostic marker, it can be used to track the inflammatory process, overall wellness, and individual’s quality of life. Lifestyle changes such as cessation of smoking, increasing physical activity, reducing body mass index led to a decrease in serum CRP levels *(Kao, Shiesh, and Wu 2006) *. Yoga and other mind-body techniques reduce inflammation through a reduction in CRP *(Morgan et al. 2014) *. An eight week Hatha yoga program compared to standard medical treatment was found to significantly reduce serum concentrations of interleukin 6 and CRP in two randomized controlled trials *(Pullen et al. 2008, 2010) *.*

Comment # 7:

 Qualitative analysis methods could be elaborated; the researchers might also consider the use of template thematic analysis

Response:

*Thank you for this suggestion. We have included the following description regarding the qualitative data analysis,*

*‘In-Depth Interviews and Focus Group Discussions will be audio-recorded, and field notes will be taken. English interviews will be transcribed verbatim while the interviews conducted in Marathi will be transcribed and translated into English. Bilingual team members, fluent in English and Marathi will carry out back-translation checks. Qualitative data will be analyzed using framework analysis approach. An a priori coding framework with a set of high-level themes will be developed. Lower order themes will be inductively derived from the data.* *NVivo* *9 software (QSR) will be used to code the data. Principal investigator will be primarily responsible for the data analysis and synthesis of the findings.’*

Comment # 8:

 Yoga Intervention:

 Well described and relevant to a population of pregnant women (focus on gentle asana, breath work and allowing for modifications for safety and stage of pregnancy).

Response:

*Many thanks for your positive response on the intervention description.*

Comment # 9:

 Discussion:

 The discussion of potential challenges to the successful implementation of the trial is helpful, as is intended outcomes.

Response:

*Thank you so much for this feedback on the discussion section.*

# Peer review: Reviewer response for version 1

**DOI:** 10.21956/wellcomeopenres.19619.r51079

**Published:** 2022-06-17

**Authors:** Carolina Estevao

**Affiliations:** 1 Kings College London, London, UK

Relevant and timely paper, an under-explored research field that could benefit from further studies. Would recommend indexing once the following comments have been addressed:

- Ensure the font used is consistent throughout – headings use a different font at the moment.

**Abstract section:**

- “a huge proportion” – please give the exact figure  and reference
- “Change in the scores…at 3 months” do you mean from baseline to 3 months?

**Background:**

- “as on 11 February 2022” – do you mean “as of” and please give an updated June 2022 figure.
- The initial paragraph should be condensed as this is the context but not the main motivation/relevance of the study protocol; could even be removed almost entirely. You may even look at the broader implications of this type of intervention for immunity for pregnant women, outside of a pandemic.

**Methods:**

- Explain Anganwadi workers and centres: community healthcare workers? Primary care? Midwives?
- Exclusion criteria – “Receiving treatment for depression or any other mental health condition” why is this an exclusion criterion? Yoga would absolutely be beneficial.
- “Five millilitres of blood will be collected at each time-point to measure the level of serum C-Reactive Protein” – please list each timepoint. Also, justify your interest in analysing CRP in the introduction, not just on page 9.
- Table 1 contains several twisted asanas and other asanas that may be unsuitable for pregnant women. How will this be adapted?
- Give details on the closing prayer.
- “they will be encouraged to undertake regular physical activity (150 min/week).” – why has this regimen been selected? How will you measure the type of activity/duration to ensure it’s uniform across the control?
- “Full compliance is defined as attendance at three or more of the first five sessions and at least four other sessions.” Are home sessions not accounted for in compliance?

Is the study design appropriate for the research question?

Yes

Is the rationale for, and objectives of, the study clearly described?

Yes

Are sufficient details of the methods provided to allow replication by others?

Yes

Are the datasets clearly presented in a useable and accessible format?

Not applicable

Reviewer Expertise:

Perinatal mental health, community-based interventions, low-income population research, yoga, psychiatry, psychology

I confirm that I have read this submission and believe that I have an appropriate level of expertise to confirm that it is of an acceptable scientific standard, however I have significant reservations, as outlined above.

# Response

**Published:** 2022-07-21

**Authors:** Rahul Shidhaye

**Affiliations:** Pravara Institute of Medical Sciences , India

**21 July 2022**

 Dear Dr. Estevao,

**Re: Manuscript title: Yoga to improve maternal mental health and immune function during the COVID-19 crisis (Yoga-M2 trial): study protocol for a pilot randomized controlled trial.**

 Many thanks for taking out time to review our submission and providing very valuable comments and suggestions. We have responded to each of your comments in italics below and believe we have been able to address all the comments. We have made corresponding changes to the manuscript and the revised manuscript is uploaded.

Comment # 1:

 Relevant and timely paper, an under-explored research field that could benefit from further studies. Would recommend indexing once the following comments have been addressed.

Response:

*Thank you so much for your positive feedback.*

Comment # 2:

 Ensure the font used is consistent throughout – headings use a different font at the moment.

Response:

*We agree with your suggestion, however, the font used for the headings is selected by the editorial team at the Wellcome Open Research. We will leave it to them to decide about the use of fonts in the final version.*

Comment # 3:

 Abstract section:

 “a huge proportion” – please give the exact figure  and reference

Response:

*Prevalence of depression during pregnancy is included in the background section in the main text. We agree that the use of the term, ‘huge proportion’, sounds vague, so have modified this sentence as follows,*

*‘Stress and depression during pregnancy negatively impact birthweight and neuro-cognitive development of the fetus.’*

Comment # 4:

 “Change in the scores…at 3 months” do you mean from baseline to 3 months?

Response:

*We have modified this sentence as follows,*

*‘Change in the scores of the Perceived Stress Scale (PSS), EuroQoL 5 Dimensions Score (EQ-5D-5L), Wisconsin Upper Respiratory Symptom Severity Scale (WURSS-21), and serum C-Reactive Protein at 3 months from baseline will be used to assess preliminary efficacy.’*

Comment # 5:

 Background:

 “as on 11 February 2022” – do you mean “as of” and please give an updated June 2022 figure.

Response:

*Please see response to comment # 6*

Comment # 6:

 The initial paragraph should be condensed as this is the context but not the main motivation/relevance of the study protocol; could even be removed almost entirely. You may even look at the broader implications of this type of intervention for immunity for pregnant women, outside of a pandemic.

Response:

*We agree with this suggestion and have revised the introduction as follows,*

*‘Pregnancy is a period of increased vulnerability for women. Changes in cardiorespiratory and immune system functioning increase the susceptibility of women to severe infection and hypoxic compromise* *(1)* *. This is particularly important as the global crisis due to the coronavirus disease (COVID-19) enters the third year. COVID-19 during pregnancy increases the risk of preeclampsia, venous thromboembolism, premature fetal membrane rupture, or preterm delivery* *(2,3)* *. In addition to increased susceptibility to infections, pregnant women are also more vulnerable to psychological stress. Mental health conditions affect pregnant women, especially depression which is quite common during perinatal period. O* *ne in four women in South Asia is likely to experience depression during pregnancy,* *while in India, the pooled prevalence of antenatal depression is 17.7% (95% CI: 11.2%* *–* *26.9%)* *(4)* *.* *Antenatal depression is associated with higher risk of pregnancy-related mortality and morbidity, pre-term delivery, low birth-weight, post-partum depression and adverse neuro-cognitive developmental outcomes in infants, children and adolescents* *(5)* *.*

*Yoga is a mind-body practice, and its roots can be traced in ancient Indian philosophy. Yoga has been practiced in India for more than 7000 years* *(6)* *. Yoga during pregnancy can be very helpful in* *prevention and treatment of stress and depression* *(7)* *. A meta-analysis found a positive effect of yoga on antenatal depression* *(8)* *. Another systematic review assessing the effect of yoga on immune system functioning found that yoga can down regulate pro-inflammatory markers and enhance cell-mediated and mucosal immunity. This makes yoga a suitable intervention for individuals suffering from diseases with inflammatory component and those who are at higher risk of getting infections* *(9)* ***.** There is only one study which has assessed the effect of yoga on immune function during pregnancy* *(10)* *. In this study, yoga-based intervention, which included postures, deep breathing, guided imagery, and deep relaxation was delivered to 48 women during pregnancy, 70 minutes per session, 2 sessions per week for 20 weeks. Pregnant women who practiced yoga had higher immunoglobulin A levels and lower salivary cortisol levels immediately after a yoga session, compared to those who did not practice yoga. In the long-term, salivary immunoglobulin A levels were significantly higher in the yoga group compared to the control group* *(10)* *.*

*How to assess the effect of yoga on inflammatory process is a key question. C-Reactive Protein (CRP) is a nonspecific maker of inflammatory process, the serum levels increase with body insult, tissue damage, aging, and cardiovascular diseases. Bacterial (or viral, fungal) infections lead to a marked increase in serum CRP levels. Although it is not a diagnostic marker, it can be used to track the inflammatory process, overall wellness, and individual’s quality of life. Lifestyle changes such as cessation of smoking, increasing physical activity, reducing body mass index led to a decrease in serum CRP levels* *(11)* *. Yoga and other mind-body techniques reduce inflammation through a reduction in CRP* *(12)* *. An eight week Hatha yoga program compared to standard medical treatment was found to significantly reduce serum concentrations of interleukin 6 and CRP in two randomized controlled trials* *(13,14)* *.*

***Rationale for study***

*The possibility of another COVID-19 wave in India (and other parts of the globe) can’t be entirely ruled out. This necessitates generating evidence for population-based interventions which can improve immune system functioning and reduce the susceptibility of infections in vulnerable groups like pregnant women. Yoga can be practiced by pregnant women as self-care with minimal training. However, there are no studies from India which have assessed the effects of yoga-based intervention on immune system functioning in pregnant women.’*

 Methods:

Comment # 7:

 Explain Anganwadi workers and centres: community healthcare workers? Primary care? Midwives?

Response:

*Thank you for this suggestion. We have added the following information about the Anganwadi centres and Anganwadi workers on page 3,*

*‘* *Anganwadi* *is a community-based center providing healthcare check-up and referral health services, supplementary nutrition, immunization, and non-formal pre-school education. Expectant and nursing mothers, children, and adolescent girls are the primary beneficiaries. These centers were established as part of the Integrated Child Development Service (ICDS) and are managed by female community health workers called Anganwadi workers. Each center caters to a population of approximately 1000 people in rural areas. Anganwadi workers have an updated list of all pregnant women in their respective areas.’*

Comment # 8:

 Exclusion criteria – “Receiving treatment for depression or any other mental health condition” why is this an exclusion criterion? Yoga would absolutely be beneficial.

Response:

*We agree with the reviewer that Yoga would be beneficial for women with depression. If we include pregnant women who are receiving treatment for depression, then we may not be able to establish if the change in the perceived stress score or the quality of life is due to the ongoing treatment for depression or due to the Yoga-based intervention. As a result, we decided to exclude women currently receiving treatment for depression.*

Comment # 9:

 “Five millilitres of blood will be collected at each time-point to measure the level of serum C-Reactive Protein” – please list each timepoint. Also, justify your interest in analysing CRP in the introduction, not just on page 9.

Response:

*We have modified the sentence as follows,*

*‘Five milliliter of blood will be collected at baseline and at three-month follow-up to measure the level of serum C-Reactive Protein.’*

*The justification for assessment of CRP in now included in the introduction.*

Comment # 10:

 Table 1 contains several twisted asanas and other asanas that may be unsuitable for pregnant women. How will this be adapted?

Response:

*All asanas included in the Yoga-M _2_ intervention (e.g., Vakrasan/half spinal twist pose) were earlier included in the Integrated Approach for Yoga Therapy during pregnancy (IAYT-P) intervention. This intervention was earlier evaluated using a randomized controlled trial design in an urban population of pregnant women in Southern India* *(Satyapriya et al., 2013)* *.*

*These asanas, including the twisted asanas are also included in a guideline document titled, ‘Yoga for Pregnant Ladies’, by the Ministry of Women and Child Development and Ministry of AYUSH (Ayurveda, Yoga, Unani, Siddha, Homeopathy), Government of India. This document is available online* *http://www.icdsbih.gov.in/PoshanAbhiyan/Guideline/YogaGuideline/Yoga_for_Pregnant_Ladies_English.pdf*

*One of the co-authors (Dr. Hemant Bhargav) is Assistant Professor of Yoga in the Department of Integrative Medicine, National Institute of Mental Health and Neurosciences, Bengaluru, India. He has led several studies on yoga-based interventions in the past. He had reviewed all the asanas before they were included in the protocol.*

*Another co-author, Dr. Vidyadhar Bangal is Professor and Head of the Department of Obstetrics and Gynaecology in the Pravara Institute of Medical Sciences. He has also reviewed the intervention protocol in detail.*

*We also conducted two intervention adaptation workshops with a total of seven pregnant women. In these workshops, feedback of women on each of the asanas was obtained. None of them said that it is not possible to perform any of the asanas, so the yoga sequence was not changed.*

*During the trial implementation, we will inform pregnant women in Yoga-M _2_ group that they should perform only those asanas which they find comfortable. We will collect the information about this and report in the findings paper.*

Comment # 11:

 Give details on the closing prayer.

Response:

*The closing prayer included in the protocol is,*

*‘Om, Sarve Bhavantu Sukhinah,*

*Sarve Santu Nirāmayāh*

*Sarve Bhadrāni Paśyantu,*

*Mā kascit Duhkha Bhāgabhavet*

*Om, Sāntih Sāntih Sāntih’*

*The meaning of this ‘Shanti Path’ (peace recital) in English is,*

*‘May All become happy,*

*May All be free from illness,*

*May All see what is auspicious,*

*May No one suffer,*

*Om peace, peace, peace.’*

*Yoga session concludes with this prayer/shanti path.*

*We have now included this closing prayer in table 1.*

*We have also included the following opening prayer in table 1,*

*‘Om, Sahana Vavatu Sahana Bhunattu*

*Saha Viryam Karawavahai*

*Tejasvinavaditamastu*

*Ma vidvishavahai*

*Om, Sāntih Sāntih Sāntih’*

*The meaning of this prayer in English is,*

*‘Together may we be protected,*

*Together may we be nourished*

*Together may we work with great energy,*

*May our journey together be brilliant and effective,*

*May there be no bad feelings between us*

*Om peace, peace, peace.’*

Comment # 12:

 “they will be encouraged to undertake regular physical activity (150 min/week).” – why has this regimen been selected? How will you measure the type of activity/duration to ensure it’s uniform across the control?

Response:

*Pregnant women in the Enhanced Usual Care (EUC) arm will be advised to undertake regular physical activity based on the recommendation by the American College of Obstetrics and Gynaecology opinion document number 804. The full citation of this is below,*

*Physical Activity and Exercise During Pregnancy and the Postpartum Period: ACOG Committee Opinion, Number 804. Obstet Gynecol. 2020 Apr;135(4):e178-e188. doi: 10.1097/AOG.0000000000003772. PMID: 32217980.*

*As mentioned in the protocol, pregnant women are neither screened for stress nor is there any detailed discussion about lifestyle during pregnancy (diet, sleep, physical activity). Participants in the EUC arm will be screened for stress, they will be referred to psychiatrist if they have moderate stress and will receive a single health education session as an enhancement over the existing care provided in the antenatal clinic. Beyond this there is no plan to monitor if they follow the suggestions related to sleep hygiene, diet, and physical activity.*

Comment # 13:

 “Full compliance is defined as attendance at three or more of the first five sessions and at least four other sessions.” Are home sessions not accounted for in compliance?

Response:

*The estimation of home practice of yoga will be based on self-reported logbooks. As this is likely to introduce information bias, we have not included this in the definition of compliance. We will however report the duration of home practice in the findings of the trial.*
